# Live Usability Testing of Two Complex Clinical Decision Support Tools: Observational Study

**DOI:** 10.2196/12471

**Published:** 2019-04-15

**Authors:** Safiya Richardson, David Feldstein, Thomas McGinn, Linda S Park, Sundas Khan, Rachel Hess, Paul D Smith, Rebecca Grochow Mishuris, Lauren McCullagh, Devin Mann

**Affiliations:** 1 Donald and Barbara Zucker School of Medicine at Hofstra/Northwell Hempstead, NY United States; 2 University of Wisconsin School of Medicine and Public Health Madison, WI United States; 3 School of Medicine University of Utah Salt Lake City, UT United States; 4 Boston University School of Medicine Boston, MA United States; 5 New York University School of Medicine New York, NY United States

**Keywords:** usability, usability testing, user experience, clinical decision support, health informatics, provider adoption, workflow, live usability, clinical prediction rules

## Abstract

**Background:**

Potential of the electronic health records (EHR) and clinical decision support (CDS) systems to improve the practice of medicine has been tempered by poor design and the resulting burden they place on providers. CDS is rarely tested in the real clinical environment. As a result, many tools are hard to use, placing strain on providers and resulting in low adoption rates. The existing CDS usability literature relies primarily on expert opinion and provider feedback via survey. This is the first study to evaluate CDS usability and the provider-computer-patient interaction with complex CDS in the real clinical environment.

**Objective:**

This study aimed to further understand the barriers and facilitators of meaningful CDS usage within a real clinical context.

**Methods:**

This qualitative observational study was conducted with 3 primary care providers during 6 patient care sessions. In patients with the chief complaint of sore throat, a CDS tool built with the Centor Score was used to stratify the risk of group A Streptococcus pharyngitis. In patients with a chief complaint of cough or upper respiratory tract infection, a CDS tool built with the Heckerling Rule was used to stratify the risk of pneumonia. During usability testing, all human-computer interactions, including audio and continuous screen capture, were recorded using the Camtasia software. Participants’ comments and interactions with the tool during clinical sessions and participant comments during a postsession brief interview were placed into coding categories and analyzed for generalizable themes.

**Results:**

In the 6 encounters observed, primary care providers toggled between addressing either the computer or the patient during the visit. Minimal time was spent listening to the patient without engaging the EHR. Participants mostly used the CDS tool with the patient, asking questions to populate the calculator and discussing the results of the risk assessment; they reported the ability to do this as the major benefit of the tool. All providers were interrupted during their use of the CDS tool by the need to refer to other sections of the chart. In half of the visits, patients’ clinical symptoms challenged the applicability of the tool to calculate the risk of bacterial infection. Primary care providers rarely used the incorporated incentives for CDS usage, including progress notes and patient instructions.

**Conclusions:**

Live usability testing of these CDS tools generated insights about their role in the patient-provider interaction. CDS may contribute to the interaction by being simultaneously viewed by the provider and patient. CDS can improve usability and lessen the strain it places on providers by being short, flexible, and customizable to unique provider workflow. A useful component of CDS is being as widely applicable as possible and ensuring that its functions represent the fastest way to perform a particular task.

## Introduction

### Background

The landmark Institute of Medicine report *To Err Is Human*, sparked an increased focus on the prevention of medical errors [1]. Computerized clinical decision support (CDS) aids providers in clinical decision making for individual patients [2] and was proposed as a key tool to improve quality of care by providers, policy makers, experts, and consumers [1,3,4]. In the United States, unprecedented resources were committed to support the adoption and use of electronic health records (EHRs) through the Health Information Technology for Economic and Clinical Health Act (HITECH) of 2009 including incentive payments by the federal government totaling up to US $27 billion over 10 years [5]. EHR adoption in eligible hospitals and practices grew from less than 10% in 2008 to over 80% in 2015 [6]. One of the HITECH requirements, for meaningful use of EHRs, included criteria to implement CDS at every stage.

CDS can improve quality by improving diagnosis, treatment, and preventative care services [7-20], but it now contributes to the increasing complexity of clinical practice. Murphy et al reported primary care doctors receive 77 notifications in the EHR per day [21] and spend nearly 2 hours on the EHR and desk work for every hour of face-to-face time with their patients [22]. Poor EHR usability is a major driver of declining career satisfaction among providers [23]. CDS is almost never tested in real clinical care sessions that have real-time pressure and patient-case complexity. As a result, many tools that appear usable and useful during development and usability testing, are cumbersome within workflow, are poorly adopted, and fail to deliver on their promise of improved care [14].

There is an extensive literature detailing the features of highly usable CDS. The foundational article “Ten Commandments for Effective Clinical Decision Support” specifies the importance of creating CDS that is fast, anticipates provider needs, fits into user workflow, provides a change in practice as opposed to a stop, is simple with few user inputs, and is adaptive [24]. A comprehensive literature review of studies evaluating barriers to and facilitators of CDS usage details similar CDS-specific usability issues including minimal mouse clicks and workflow integration [25]. These works and many others [26-33] are important but primarily based on expert opinion and provider feedback given via surveys, interviews, and simulated usability testing. Few have objectively observed providers during a real clinical session and none has observed the provider interaction with complex CDS.

### Objectives

The objective of this study was to further understand the barriers to and facilitators of meaningful CDS tool usage within a real clinical context. Usability testing of 2 CDS tools was conducted as a part of the study “Integrated Clinical Prediction Rules: Bringing Evidence to Diverse Primary Care Settings (iCPR2),” a randomized controlled trial evaluating the tools’ effect on antibiotic ordering [34]. The CDS tools were composed of an alert, a clinical prediction rule (Centor Score and Heckerling Rule) estimating risk of either group A Streptococcus (GAS) pharyngitis or pneumonia, and an automatic order set based on risk.

## Methods

This was a qualitative observational study done in January 2017 at the University of Wisconsin-Madison, School of Medicine, a large academic health care center, where the parent study was being conducted. Testing was completed with a convenience sample of 3 volunteer primary care providers during a total of 6 patient care sessions. Inclusion criteria required that participants (1) worked in Family Medicine or Internal Medicine clinics, (2) spent at least half of their time providing clinical care, and (3) were randomized to the intervention arm of the larger Integrated Clinical Prediction Rules: Bringing Evidence to Diverse Primary Care Settings (iCPR2) study with CDS embedded in their EHR system. The sample size was typical for usability studies and was considered sufficient to elicit the vast majority of usability issues [35-37]. The sample size was considered to be 6, for each patient care session, as each was a complex and unique interaction between the patient, provider, and CDS tool. A typical sample size for usability studies is 5.

**Figure 1:**
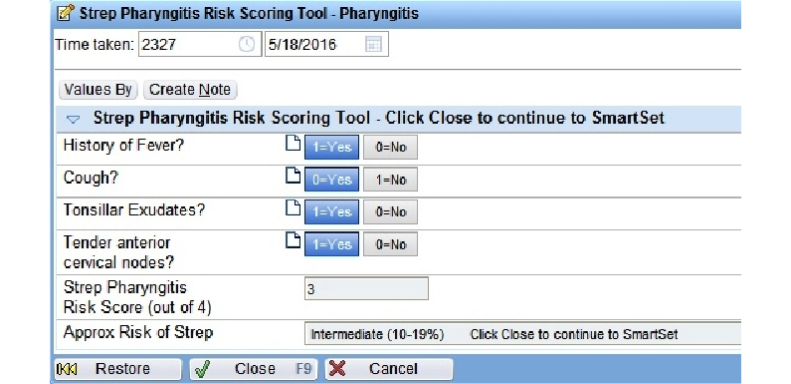
Clinical decision support tool calculator.

**Figure 2:**
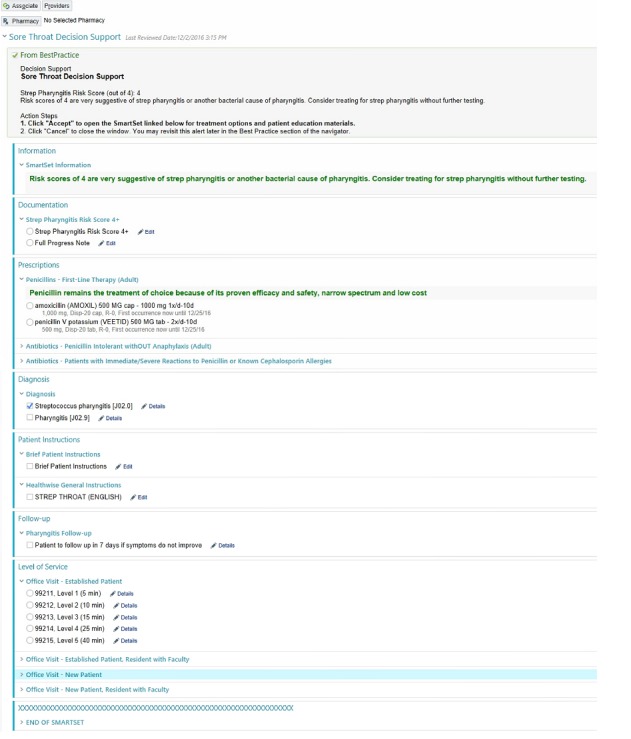
Clinical decision support tool automatic order set.

The 2 CDS tools tested in the parent study used clinical prediction rules to evaluate the risk of GAS pharyngitis in patients presenting with sore throat (the Centor Score) and the risk of pneumonia in patients presenting with cough or upper respiratory tract infection (the Heckerling Rule). The tools were both built in the EpicCare ambulatory EHR (Epic Corp. Verona, Wisconsin). The tools were triggered by a reason for visit of sore throat, cough, or upper respiratory tract infection. When triggered, the provider was presented with an alert offering the CDS tool upon opening the chart. If accepted, the provider was taken to a calculator with a list of clinical questions, each of which contributes to a total risk score (Figure 1). After calculator completion, the provider was shown a risk score, identifying the patient as low, intermediate, or high risk for the condition as well as offered an order set tailored to the calculated risk. These order sets included documentation for progress notes, laboratory orders, prescription orders, diagnoses, patient instructions, and level of service (Figure 2).

Live usability testing was conducted in a clinical office setting. Written informed consent was obtained from all participating providers the day before the study observations. At that time, the study procedures were reviewed with the providers and their staff. Testing was performed for 1 day for each of the providers. On the day of live usability testing, the providers’ receptionist handed out a flyer with details about the study to all of the participating providers’ patients. Study staff approached these patients to ask if they were being seen for a cough, sore throat, or an upper respiratory tract infection. Patients with these symptoms were provided with an explanation of the study and verbal consent was obtained.

All human-computer interactions, including audio and continuous screen capture, were recorded using Camtasia (TechSmith, Okemos, MI, USA) software. Before the start of the patient care session, the usability testing software was set to record. It was paused if patients left the room for testing and stopped at the end of the visit. After the provider’s care sessions were completed, they were briefly interviewed about their general attitudes toward the tool. These interviews were recorded using a digital voice recorder.

All provider and patient verbalizations from the visits and the interviews were transcribed verbatim. The video from the visits, audio from the interviews, and the transcriptions of both underwent thematic analysis and were coded using the following process: a total of 2 coders used a triangulation approach involving iteratively watching the videos, listening to the interviews, and reading the transcriptions. This allowed a broader and more complex understanding of the data attained. Those 2 coders then undertook development of a codebook reflecting the emerging themes with no a priori codes used. Using the constant comparative method, additional readings of the transcription led to the consolidation of these coding schemes until no further refinement was required. The primary themes identified were: Tool Interruptions, Workflow, Tool Applicability, Patient-Tool interaction, Provider-Computer- Patient Interaction, Ease of Use, and Missed Opportunities. Transcribed audio from the visit and the interview along with observed participant interaction with the tool were coded by hand and were categorized under each code by 2 independent coders and analyzed for themes that would be generalizable to most CDS. The themes were reviewed together by the coders, and all discrepancies were resolved by discussion to achieve a consensus leading to 100% agreement between the coders. This was formative as opposed to summative usability testing. We did not measure task times, completion rates, or satisfaction scores. The institutional review board at the University of Wisconsin approved the research protocol.

## Results

### Overview

All 3 participants were primary care providers: 2 nurse practitioners and 1 medical doctor. There were a total of 6 patient encounters. Although 5 of these were acute or follow-up visits that lasted about 15 min each, 1 was a complete physical exam that was about 30 min in length. In half of the visits, the patients presented with the chief complaint of sore throat, and the CDS tool built with the Centor Score was used to stratify the risk of GAS pharyngitis. In the other half of the visits, the patients presented with a chief complaint of cough or upper respiratory tract infection, and the CDS tool built with the Heckerling Rule was used to stratify the risk of pneumonia. As the tools were so similar, with the exception of clinical content, they were analyzed together. Example visit quotes, participant actions, and participant interview quotes are included in Table 1 by coding category along with a summary and recommendations for future CDS.

**Table 1:** Live usability testing results.

Coding category, example comments or actions^a^		Summary and recommendation
**Tool interruptions**		
	Patient: “Was it last year or the year before – didn’t I have to get a pneumonia shot?” *Provider navigates away from automatic order set immediately after opening it.*	During every testing session, the provider was interrupted during their use of the CDS^b^ tool by the need to refer to other sections of the chart.
	Provider: “Have you had a chest X-ray anytime recently?” *Provider clicks away from automatic order set to review results of last CXR*^c^.	Recommendation: Complex CDS should be built for disrupted workflow, with easy and obvious re-entry points.
**Workflow**		
	*Provider opens chart, clicks away from alert, to progress notes*.	During every testing session, the progress note served as the center point of the provider interaction with the electronic health record.
	“It’s the first thing that comes up...but you have to get all that info from the patient first. So that’s what I mean by clunky.” [PCI^d^]	—^e^
	At the start of visit, all providers navigate immediately to the progress note. Half of them spent more than 95% of the visit with this function open, and only 1 spent more than 40% of the visit time with it open. [QM^f^]	Recommendation: CDS tools that exist within the progress note may have higher adoption rates because it would be more likely they were present at the time of decision making.
**Tool applicability**		
	Provider: “So I read your chart; it says that you’ve been having symptoms as deer season?”	In half of the sessions, patient history challenged the validity of the clinical prediction rule used to calculate risk.
	Patient: “I actually called in and Dr. [name] gave me a prescription...”	—
	“Sometimes...something in your clinical encounter still says, 'get the X-ray or still treat,' you know, maybe you saw them before.” [PCI]	Recommendation: CDS tools should be as broadly applicable as possible with clear indications for use.
**Patient-tool interaction**		
	Provider: “OK, so our little risk calculator here is recommending that we would swab you for strep throat, and I agree with that.”	In every session in which the tool was used to assess risk, the provider completed the calculator with the patient.
	Provider: “But your heart is beating kinda fast, you’ve had a fever last night...the recommendation would be to get a chest x-ray today.”	—
	“I like to be able to show it to patients. So that part of it I really – I like to have that support, and that extra backup for the decision that I want to make.” [PCI]	Recommendation: CDS tools should be designed to be viewed by the patient and provider simultaneously.
**Provider-computer-patient interaction**		
	Patient: “My brother’s living with me, he’s a vet...” *Provider enters data from chart review into progress note while patient is talking about something unrelated.*	In every testing session, the providers toggled between addressing either the computer or the patient during the visit.
	Provider: “So basically to summarize: about 9 days ago is when you first got sick...” *Physician stops interacting with computer to recap history.*	—
	(Silence while physician types)	—
	Providers spent 0% to 3% of their visit time listening to the patient without simultaneously engaging with the computer. [QM]	Recommendation: Providers may find CDS tools easier to complete if they engage patients.
**Ease of use**		
	Provider: “Hold on, I just need the laboratory to actually put in the results... my thing isn’t popping up for me to prescribe the antibiotics quite yet.”	Providers were able to complete the tool quickly; however, during half of the sessions, hard stops and fixed elements in the tool created barriers to usability.
	“The patient instructions have some hard stop, so I got frustrated with that, and then eventually deleted and typed my own patient instructions in.” [PCI]	—
	“Cause it’s short. If it were any longer, I’d probably get frustrated with it.” [PCI]	—
	Providers spent about 1 min of the visit time completing the CDS tool. [QM]	Recommendation: Tools that are short, customizable, and flexible to different workflows will have improved usability.
**Missed opportunities**		
	*Provider enters shortcut “.cvuri” to generate upper respiratory infection note template at start of visit.*	In every session, providers did not use either the automatic order set or automatic documentation.
	Provider: “So the antibiotic that I would pick for you is one called Azithromycin.” *Provider orders antibiotics a la carte without re-entering tool after chest x-ray is resulted*.	—
	“It’s easier for me to order a chest X-ray just outside of the order set...then get the results back and go on with the patient visit. And then at that point, it’s like the opportunity has been lost to use the [automatic order] set.” [PCI]	Recommendation: Elements that are incorporated into CDS tools as incentives should save the provider time or effort when compared with their usual workflow.

^a^Provider and patient statements during the visit are included in quotations, and provider actions are in italics.

^b^CDS: clinical decision support.

^c^CXR: chest x-ray.

^d^PCI: provider comments during interview.

^e^The Summary and Recommendation for each of the Coding Categories applies to all of the data provided.

^f^QM: quantitative measurements.

**Figure 3:**
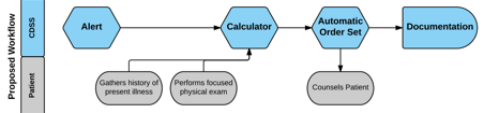
Clinical decision support system proposed workflow.

### Coding Categories

#### Tool Interruptions

Although the tool was built to be completed sequentially and without interruption (Figure 3), all participants were interrupted during their use of the CDS tool. Participants were typically triggered to navigate away from the CDS tool by questions that came up during the encounter about the patient’s previous medical history (eg, vaccine record and laboratory test results). Each of these deviations required the participant to remember to navigate back to the CDS tool and to know how to do this.

#### Workflow

Upon opening the chart, every participant was taken to an alert for the CDS tool. At the start of each patient session, the provider navigated away from the alert to the progress note and began taking the history of present illness. During most patient sessions, the provider then completed the physical exam, brought the patient back to the computer, and engaged with the CDS tool. The progress note served as the center point of the participant interaction with more than 95% of visit time spent with the progress note feature open in half of the sessions.

#### Tool Applicability

In half of the patient visits, patients reported some piece of information, typically as a part of the history of present illness that raised a question for the coders of whether the tool was applicable to their clinical condition. For example, 2 of the patient encounters were for complaints consistent with sinusitis and 1 patient with cough had been previously treated. All of the providers in the postsession brief interviews mentioned the value of a more broadly applicable tool that included CDS for bacterial sinusitis. They felt that this addition would allow them to use the tool more often.

#### Patient-Tool Interaction

A majority of the providers used the tool to assess risk by showing the patients the tool while they completed it and explained the results of the calculator to the patient. They all reported that the ability to show the patient their risk of a bacterial infection was the strongest feature of the tool. Providers reported using the tool to educate patients about their risk and manage patient expectations more than using it to discover the patient’s risk of bacterial infection.

#### Provider-Computer-Patient Interaction

Providers spent most of the visit either talking to the patient or interacting with the EHR. They spent 0% to 3% of their time listening to the patient without engaging the EHR. For example, to gather the history of the present illness, providers typically started with an open-ended question. As the patient began talking, they shifted their focus to the EHR to begin typing the progress note. They took the opportunity to review the chart if the patient began talking about unrelated topics. At times when the patient was not speaking but the provider needed to interact with the EHR (eg, completing orders at the end of the visit), there would be silence.

#### Ease of Use

Providers commented on the tool’s brevity as being a significant strength, making it easier to use. They spent about 1 min of the patient visit completing the tool. Hard stops and fixed elements within the tool led to frustrations. For example, after a verbal communication about a positive rapid GAS pharyngitis result, the provider could not continue to the automatic order set until the result was properly registered by the laboratory, requiring the provider to leave the patient, go back to the laboratory, and resolve the issue before continuing with the patient visit.

#### Missed Opportunities

Although the tool was designed to automatically generate visit documentation as an incentive for tool completion, every provider started writing his or her note at the beginning of the visit. Each provider used shortcuts to template their notes, which increased the comparative ease of use of typing their note without using the tool’s feature. Although the tool’s automatic order set was also designed as an incentive for use, participants described it being easier to order antibiotics and tests outside of it.

## Discussion

### Principal Findings

This study contributes to our growing understanding of how to develop usable and useful CDS tools, particularly considering the provider-computer-patient interaction. This study builds on our previous work analyzing results from the “Think Aloud” and “Near Live” usability testing of these 2 CDS tools [38]. Each of these 3 types of usability testing generated unique and generalizable insights. As testing increasingly approached reality, additional types of barriers to and facilitators of CDS usage were found. During the “Think Aloud” testing, providers were presented with a written clinical case while interacting with the tool. Commentary focused on improving the ease of use of the tool. During the “Near Live” testing, providers interacted with a patient actor and commentary addressed ease of use of the tool with an added, more focused evaluation of its usefulness. Previous studies have also found that as usability testing approaches reality, themes and insights shift from mostly surface-level ease–of-use issues to high-level usefulness and workflow issues [28]. Live usability testing provided insights on the tools’ ease of use, usefulness, and impact on the patient-provider interaction that were not evident in previous usability testing.

#### Provider-Computer-Patient Interaction and Patient-Tool Interaction

Our observation of the minimal time providers spent listening to the patient without simultaneously interacting with the computer speaks to the growing demands of the EHR. Each of these demands must take the place of some part of what was already a full visit. In a typical encounter, a provider listens to the patient, examines the patient, and talks to the patient. The pressure to “multitask” using the EHR is easiest while listening to the patient. Notably, however, there is evidence that providers are doing this without decreasing patient satisfaction or diminishing the patient-provider relationship [20]. The use of EHRs in the ambulatory setting also does not seem to decrease quality of care [39]. However, the EHR contains a wealth of information that has the potential to positively impact care. The simple, intuitive, and informational design of this tool allowed providers to use it with their patients, allowing the EHR to provide important information while reconnecting the patient and the provider.

CDS designers have largely focused on these tools’ contribution to medical decision making without considering its collaborative nature. To varying degrees, every medical decision is a shared decision. CDS tools that are built to engage both patient and provider target both decision makers. Every provider in this study cited the ability to share the tool’s results with the patient as its greatest strength. These providers did not need a better understanding of patient’s risk of bacterial infection as much as they needed a better way to communicate this information to the patient. CDS that accounts for the patient’s role in decision making may be used to facilitate shared decision making, which may improve usability, increase adoption rates, thereby resulting in improved quality of care.

#### Tool Interruptions, Usability, and Workflow

The expected workflow for the tool was not observed in any encounter, and the providers did not use the tool at the time it triggered. In addition, when the tool was used, they were unable to flow from alert to calculator to automatic order set as it was designed to be used. These findings point to the existence of significant provider workflow variability. Primary care provider workflow is not prespecified and emerges based on the unique interaction between the patient and the provider’s agendas [40]. Our study points to a short, flexible, and customizable CDS tool as more usable. Locating the CDS inside the progress note may help to address tool interruptions and improve usability and workflow. The progress note seems to be the center point of provider interaction with the computer. For many providers, this would make the tool available at the time of decision making and present while they use the split screen to refer back to the chart when necessary.

#### Missed Opportunities and Tool Applicability

The ability to use the tool in as many clinical situations as possible increases its usefulness. Every provider commented on the utility of adding a tool addressing risk of bacterial sinusitis. This addition would allow providers to apply these tools to almost any symptoms of upper respiratory tract infection. The more broadly these tools apply, the more valuable they may be to providers. In half of the visits, patient history challenged the validity of the clinical prediction rule used to calculate the risk of bacterial infection. Usefulness was addressed as well with providers’ lack of use of the incorporated incentives. Elements that are incorporated into CDS tools as incentives should save the provider time or effort when compared with their usual workflow. The lack of order set use can also limit the ability of the CDS to improve evidence-based patient care and influence the type of antibiotics ordered.

Usability testing of CDS helps to close the gap between its current and potential impact on providers, their interactions with patients, and the quality of care they give. Although the EHR’s poor usability and interference with face-to-face patient care are prominent sources of professional dissatisfaction, providers still believe in the potential of this technology [23]. The concept of evidence-based clinical care revolutionized medicine by demanding that interventions be formally evaluated. We must evaluate CDS with this same rigorous approach; usability tested and refined CDS can address unforeseen consequences, decrease strain on the provider and the patient-provider interaction, and garner the adoption rates required to have a meaningful positive impact.

### Limitations

As typical for usability studies, participants were a convenience sample of volunteers rather than a representative sample. They were identified based on their higher-than-average use of this CDS tool. This was done to ensure tool usage on the day of testing. These providers may have a more positive opinion of it or use it in a way that is fundamentally different from that of the average provider. Even in this subset of providers predisposed to high CDS use, the tool was not used as designed and created workflow frustration. These providers may also use the EHR more during patient encounters than average. The sample size for this study was small because of the inherent logistical difficulty of live usability testing in the real clinical environment. However, usability testing is typically performed in just 5 sessions as thematic saturation begins to occur at this point [35-37]. We reached thematic saturation during our study, observing consistent and recurring themes across all of our recorded sessions. During testing, participants were aware that they were being recorded and may have changed their behavior and reported observations because of being observed (the Hawthorne effect). This testing was done with just 1 EHR, EpicCare, which may limit generalizability. However, this is the most widely used EHR in the United States. All of these limitations are inherent to usability studies and represent standard practice.

### Conclusions

Live usability testing of this CDS tool provided insights on its ease of use, usefulness, and its impact on the patient-provider interactions that were not evident in previous usability testing. This highlights the importance of incorporating live usability testing into CDS tool development. Our study suggests that short, flexible, and customizable CDS tools may be more usable, addressing the challenges of the highly variable provider workflow. The progress note seems to be the center point of provider interaction with the EHR. Locating the CDS tool inside the progress note may help to address tool interruptions and ensure that the tool is available at the time of decision- making and present when providers refer back to the chart when necessary. The tool was designed to be used sequentially and this contributed to providers not finishing the tool once they deviated from the intended workflow.

The more broadly these tools apply, the more valuable they are to providers. Elements that are incorporated into CDS tools as incentives must be useful, saving the provider time or effort when compared with their usual workflow. Live usability testing of these tools also generated insights about their impact on the patient-provider interaction. The simple, intuitive, and informational design of the tool allowed providers to use it with their patients. CDS can contribute to the patient-provider interaction by being built to be simultaneously viewed by the provider and patient. The use of the calculator to engage the patient in the decision-making process as a driver for the use of the CDS tool needs further study. This allows the EHR to provide important information while reconnecting patient and provider.

## Abbreviations

CDS: clinical decision support
EHR: electronic health record
GAS: group A Streptococcus
HITECH: Health Information Technology for Economic and Clinical Health Act
